# *GST M1-T1 null* Allele Frequency Patterns in Geographically Assorted Human Populations: A Phylogenetic Approach

**DOI:** 10.1371/journal.pone.0118660

**Published:** 2015-04-13

**Authors:** Senthilkumar Pitchalu Kasthurinaidu, Thirumurugan Ramasamy, Jayachitra Ayyavoo, Dhvani Kirtikumar Dave, Divya Anantray Adroja

**Affiliations:** 1 Department of Biotechnology, Shree M. & N. Virani Science College, Rajkot, Gujarat, 360 005, India; 2 Department of Plant Biotechnology, School of Biotechnology, Madurai Kamaraj University, Madurai, Tamilnadu, 625 021, India; 3 Department of Animal Science, School of Life Sciences, Bharathidasan University, Tiruchirappalli, Tamilnadu, 620 024, India; Mathematical Institute, HUNGARY

## Abstract

Genetic diversity in drug metabolism and disposition is mainly considered as the outcome of the inter-individual genetic variation in polymorphism of drug-xenobiotic metabolizing enzyme (XME). Among the XMEs, glutathione-S-transferases (*GST*) gene loci are an important candidate for the investigation of diversity in allele frequency, as the deletion mutations in *GST M1* and *T1* genotypes are associated with various cancers and genetic disorders of all major Population Affiliations (PAs). Therefore, the present population based phylogenetic study was focused to uncover the frequency distribution pattern in *GST M1* and *T1 null* genotypes among 45 Geographically Assorted Human Populations (GAHPs). The frequency distribution pattern for *GST M1* and *T1 null* alleles have been detected in this study using the data derived from literatures representing 44 populations affiliated to Africa, Asia, Europe, South America and the genome of PA from Gujarat, a region in western India. Allele frequency counting for Gujarat PA and scattered plot analysis for geographical distribution among the PAs were performed in SPSS-21. The *GST M1* and *GST T1 null* allele frequencies patterns of the PAs were computed in Seqboot, Gendist program of Phylip software package (3.69 versions) and Unweighted Pair Group method with Arithmetic Mean in Mega-6 software. Allele frequencies from South African Xhosa tribe, East African Zimbabwe, East African Ethiopia, North African Egypt, Caucasian, South Asian Afghanistan and South Indian Andhra Pradesh have been identified as the probable seven patterns among the 45 GAHPs investigated in this study for *GST M1-T1 null* genotypes. The patternized null allele frequencies demonstrated in this study for the first time addresses the missing link in *GST M1-T1 null* allele frequencies among GAHPs.

## Introduction

Metabolic activities play an important role in shaping the livelihood of an organism. Drug-xenobiotic compound metabolizing enzyme (XME) systems are the most investigated pathways that are involved in equilibrating the health status of an individual. Among the numerous drug related genes investigated, Glutathione-S-transferases (*GST*) of Phase II XMEs were found to play an important role in cellular protection and in cellular resistance to drugs by glutathione conjugation reactions. *GST* classes convert the active endogenous and/or exogenous carcinogenic compounds to their detoxified form. Among the *GST* classes, *GST M1* and *GST T1* were found associated to a loss of function with a structural deletion (*null* mutation); moreover, they were also found to modify the detoxification ability of the individual exposed to tobacco or carcinogenic pollutants in the environment [1]. Genotoxins such as aromatic hydrocarbon epoxides and products of oxidative stress such as DNA hydroperoxides, polycyclic aromatic hydrocarbon diol epoxides are catalyzed and detoxified by *GST M1* while, the constituents of cigarette smoke such as alkyl halides, bezo (a) pyrene diol epoxide, acrolein are catalyzed and detoxified by *GST T1* [1, 2]. Several factors such as environmental pollution, dietary habits and activity-dependent genetic differences have been reported as modulators of *GST* expression and susceptibility to xenobiotic compound detoxification [3]. Numerous studies in the recent past have hypothesized the difference in metabolic rate of *M1* and *T1* classes of *GST* as the risk factor associated to cancers of bladder, pancreas, upper aero digestive tract, lung, esophageal, head-neck, melanoma and also in Balken endemic nephropathy patients [4–9]. Further, the inter-individual difference in drug disposition and efficacy has been investigated by various authors [10] and the observed frequency distributions of *GST M1-T1* genotypes among different populations are reported as ethnic or PAs dependent [10, 11]. Drugs are the major hope of remedy for the people around the globe with various metabolic and genetic disorders but the scenario in the past was found distressed as the effectiveness of the drugs were reported by the influence of the unidentified polymorphic patterns observed in drug metabolism genes among different ethnics or PAs [11–13]. Though researchers from different PAs are interested in analyzing the frequencies of *GST M1* and *T1 null* genotypes and their possible risk association with various disorders, they are not able to report conclusive association in all major PAs [14, 15]. Recent advances in molecular techniques have opened a new era of pharmacogenomics and several researchers are inclined towards investigating the relationship in genetic diversity and allelic frequency of *GST* classes to insight genetic predisposition or susceptibility among various ethnics or PAs. In this context, probing the genetic variation in *GST* classes is inevitable for genomic epidemiological studies and to develop new common drugs in future to majority of PAs [9, 16, 17]. The allele frequency pattern in *GST M1-T1 null* genotypes of different PAs are yet to be explored to unlock several phenomenons related to a risk association with genetic diseases and drug dispositions [10]. A study including statistically valid number of subjects from various major PAs could address the issue of understanding the phenomenon for frequency distribution pattern in geographically assorted human populations (GAHPs); however, it will be tedious and might require huge population size [16]. Therefore, the present study was focused to uncover the genetic distance based ancestral origin or genetic affinity among GAHPs to address the paradigm for *GST M1-T1 null* allele frequency diversity. We are currently exploring how best to do this for the large number of populations in the present analysis to understand this phenomenon of frequency distribution pattern in GAHPs. *GST M1* and *GST T1* loci investigated in this study have been derived from literatures representing 44 different populations affiliated from Africa, Asia, Europe, South America and the genome of Gujarat PA, a region in western India. *GST M1*-*T1* null allele frequency of 45 GAHPs were computed for phylogenesis with pair wise genetic distance based unweighted pair group method with arithmetic mean (UPGMA) and the findings of seven patterns for *GST M1-T1 null* allele frequency in this study have been demonstrated for the first time with highest genetic affinity. The patterns of *null* allele frequencies reported in this study add insights to determine a conclusive risk association of *GST M1-T1* loci with several cancer or genetic disorders.

## Materials and Methods

### Subjects

The present investigation includes *GST M1* and *GST T1 null* allele frequency of 45 GAHPs from 39 studies. *Null* allele frequency of Gujarat population was investigated in this study from 504 healthy unrelated volunteers of Gujarati origin with a mean age of 60 years. After signing the informed consent to participate in the study, blood samples of 2 mL were collected from each subject. Data of the remaining 44 populations were collected from different populations investigated by various authors (Table 1). Several studies of same ethnicity were also gathered in the study to fulfill the statistical significance of the study and to minimize the varying frequency of polymorphism among the ethnic groups while, the data gathered from Naveen et al. [18] had allele frequencies of combined Tamilnadu and Pondicherry PAs. The study was approved by the institutional ethics committee of Shrimathi Vasantben Ratilal Desai Cancer Research Centre, Rajkot Cancer Society - India.

**Table 1:** *GST M1*-*T1 null* allele frequency of geographically assorted human populations.

	***GST M1 Null***		***GST T1 Null***		
**Geographic Region (short form)**	**Frequency**	**Sample**	**Frequency**	**Sample**	**Reference**
**I. Eastern Asia** (eAs)	**0.521**	8931	**0.476**	7875	[10]
1. Japan (eAs_Jap)	0.501	2215	0.496	1518	[10]
2. Korea (eAs_Kor)	0.527	3704	0.509	3641	[10]
3. China (eAs_Chi)	0.535	2467	0.443	2355	[10]
4. Mongolia (eAs_Mon)	0.464	207	0.256	207	[21]
**II. South Eastern Asia** (seAs)	**0.562**	1666	**0.351**	890	[10]
5. Vietnam (seAs_Vie)	0.420	100	0.300	100	[22]
6. Philippines (seAs_Phi)	0.517	60	0.333	60	[23]
7. Indonesia (seAs_Ids)	0.556	162	0.414	162	[24]
8. Singapore - Malay (seAs_S_M)	0.653	167	0.383	167	[25]
**III. Southern Asia** (sAs)					
**a. India (sAs_Ind)**	**0.296**	4720	**0.182**	4570	[14, 15, 18, 26–35], Present Study
9. Uttar Pradesh (Ind_Up)	0.327	1107	0.174	1107	[14, 29–32]
10. West Bengal (Ind_Wb)	0.270	67	0.130	67	[34]
11. Gujarat (Ind_Guj)	0.200	504	0.355	504	Present Study
12. Maharashtra (Ind_Mah)	0.299	2060	0.138	2060	26–28, 31]
13. Andhra Pradesh (Ind_Ap)	0.359	370	0.254	370	[18, 33]
14. Karnataka (Ind_Kar)	0.258	260	0.191	110	[15, 18]
15. Tamilnadu (Ind_Tn)	0.235	200	0.188	200	[18]
16. Kerala (Ind_Ker)	0.324	182	0.128	182	[18, 35]
**b. Afghanistan** (sAs_Afg)	**0.460**	656	**0.186**	656	[36]
**c. Iran** (sAs_Iran)	**0.406**	NA	**0.382**	NA	[37]
**d. Pakistan** (sAs_Pak)	**0.450**	111	**0.230**	111	[38]
**IV. Northern Europe** (nEu)	**0.533**	3686	**0.165**	2291	[10]
17. Sweden (nEu_Swd)	0.546	747	0.147	626	[5, 39]
18. Finland (nEu_Fin)	0.469	482	0.130	385	[5]
19. Denmark (nEu_Dn)	0.536	537	0.129	358	[5]
20. UK (nEu_Uk)	0.578	1122	0.205	922	[5]
**V. Western Europe** (wEu)	**0.515**	6486	**0.183**	5562	[10]
21. Netherlands (wEu_Ned)	0.504	419	0.229	419	[5]
22. Germany (wEu_Ger)	0.516	3054	0.173	3054	[4]
23. France (wEu_Fra)	0.534	1184	0.168	512	[5]
**VI. Southern Europe** (sEu)	**0.509**	3770	**0.195**	2660	[10]
24. Italy (sEu_Ita)	0.494	810	0.163	553	[5]
25. Spain (sEu_Spa)	0.504	1132	0.221	1121	[7]
26. Slovenia (sEu_Sln)	0.520	102	0.255	102	[5]
27. Greece (sEu_Gr)	0.520	171	0.099	171	[6]
**VII. Eastern Europe** (eEu)	**0.511**	1184	**0.188**	1169	[10]
28. Czech Republic (eEu_Cze)	0.567	67	0.224	67	[40]
29. Bulgaria (eEu_Bul)	0.518	112	0.161	112	[9]
30. Poland (eEu_Pol)	0.511	321	0.193	321	[41]
31. Slovakia (eEu_Slk)	0.512	332	0.180	322	[39]
32. Russia (eEu_Rus)	0.497	352	0.193	352	[42]
**VIII. Africa (Af)**					
33. North African Egypt (nAf_Egp)	0.555	200	0.295	200	[21]
34. West African Nigeria (wAf_Nig)	0.300	300	0.370	300	[43]
35. South African Xhosa (sAf_Xho)	0.211	128	0.406	128	[44]
36. South African Namibia (sAf_Nam)	0.112	134	0.358	134	[45]
37. Middle African Cameroon (mAf_Cam)	0.278	126	0.468	126	[46]
38. East African Ethiopia (eAf_Eth)	0.435	153	0.373	153	[46]
39. East African Somalia (eAf_Som)	0.400	100	0.440	100	[47]
40. East African Zimbabwe (eAf_Zim)	0.240	150	0.260	150	[48]
**IX. Caucasian** (wAs_Cau)	**0.529**	2714	**0.197**	1223	[10]
**X. South American Brazil** (sAm_Brz)	**0.397**	794	**0.267**	794	[49, 50]
**Total number of Samples**		**36009**		**29092**	

### DNA isolation and Genotyping

Lahiri and Nurnberger method was used to isolate genomic DNA from whole blood [19]; the Huang et al., method of multiplex polymerase chain reaction was performed to identify *GST M1* and *T1* polymorphism with albumin gene as internal control [20]. Amplified products of PCR were visualized in 2% agarose gel and the band patterns were analyzed for polymorphism.

### Geographically assorted human populations (GAHPs)

On the basis of interest in allele frequency patterns and availability of *GST M1-T1 null* allele frequency data shared by all populations, we choose 45 representative geographically assorted human populations around the world from 38 investigations reported by various authors from Asia [10, 14, 15, 18, 21–38], Europe [4–7, 9, 10, 39–42], Africa [21, 37, 43–50] and South America [49, 50] as summarized in Table 1. Of these 45 GAHPs, 4 were chosen from Eastern Asia (Japan, Korea, China and Mongolia [10, 21]), 4 from South Eastern Asia (Vietnam, Philippines, Indonesia and Singapore-Malay [10, 22–25]), 8 from Southern Asia India (Tamilnadu, Kerala, Karnataka, Andhra Pradesh, Maharashtra, West Bengal, Uttar Pradesh and Gujarat [14, 15, 18, 26–35, present study]), 3 from Southern Asia (Afghanistan, Iran, Pakistan [36–38]), 4 from Northern Europe (Sweden, Finland, Denmark and UK [5, 10, 39]), 4 from Southern Europe (Italy, Spain, Slovenia and Greece [5–7, 10]), 5 from Eastern Europe (Czech Republic, Bulgaria, Poland, Slovakia and Russia [9, 10, 39–42]), 3 from Western Europe (Netherlands, Germany and France [4, 5, 10]), 8 from Africa (Egypt, Nigeria, Xhosa tribe, Namibia, Cameroon, Ethiopia, Somalia and Zimbabwe [21, 37, 43–50]), 1 from South American Brazil [49, 50] and Caucasian (Americans and Canadians [10]). The “Caucasian” population used in this study was arbitrarily termed as “West Asian Caucasians” (wAs_Cau) to precise the geographical region and allele frequency patterns. All the PAs were grouped into continental regions as per the guidelines of Statistics Division of the United Nations (http://unstats.un.org/unsd/methods/m49/m49regin.htm [51]). Initially, 20 different continental region populations, as summarized in Table 2, were used in phylogenetic analysis for *GST M1*-*T1 null* allele frequency to minimize the effect of inbreeding [52]. Of these 20 continental region populations, the *null* allele frequency of thirteen populations such as Afghanistan, Iran, Pakistan (3 from South Asia), Brazil (1 from South America), Egypt (1 from North Africa), Nigeria (1 from West Africa), Xhosa tribe, Namibia (2 from South Africa), Cameroon (1 from Middle Africa), Ethiopia, Somalia and Zimbabwe (3 from East Africa), Caucasian (West Asia, as described earlier) and the average *null* allele frequency of seven continental regions such as East Asia (Japan, Korea, China, Mongolia), South East Asia (Vietnam, Philippines, Indonesia, Singapore-Malay), South Asia India (Tamilnadu, Kerala, Karnataka, Andhra Pradesh, Maharashtra, West Bengal, Uttar Pradesh, Gujarat), North Europe (Sweden, Finland, Denmark, UK), West Europe (Netherlands, Germany, France), South Europe (Italy, Spain, Slovenia, Greece) and East Europe (Czech Republic, Bulgaria, Poland, Slovakia, Russia) were used in the phylogenetic analyses, as summarized in Table 1 for *GST M1*-*T1 null* allele frequency. Since, some of the populations have considerable gene admixture from other PAs [52] another phylogenetic analysis was performed using all the 45 GAHPs for *GST M1*-*T1 null* allele frequency (**Table 3**).

**Table 2:** *GST M1-T1 null* allele frequency based genetic distance between 20 different continental region populations.

S. No.	Populations	eAf_Zim	sAf_Nam	sAf_Xho	mAf_Cam	wAf_Nig	sAs_Ind	sAm_Brz	eAf_Som	sAs_Iran	eAf_Eth	sAs_Pak	sAs_Afg	sEu	eEu	wEu	eAs	wAs_Cau	nEu	nAf_Egt	seAs
1	**eAf_Zim**																				
2	**sAf_Nam**	0.019641																			
3	**sAf_Xho**	0.018002	0.007702																		
4	**mAf_Cam**	0.036741	0.028132	0.006642																	
5	**wAf_Nig**	0.011698	0.025018	0.007576	0.009135																
6	**sAs_Ind**	0.007203	0.050393	0.046757	0.068305	0.027352															
7	**sAm_Brz**	0.019686	0.071517	0.047429	0.050077	0.017980	0.012453														
8	**eAf_Som**	0.047449	0.069856	0.031371	0.014241	0.013198	0.062987	0.027019													
9	**sAs_Iran**	0.033673	0.067958	0.033278	0.022080	0.010096	0.040405	0.011558	0.003247												
10	**eAf_Eth**	0.041147	0.083845	0.045069	0.031565	0.016495	0.043599	0.010910	0.005520	0.000882											
11	**sAs_Pak**	0.037341	0.107807	0.078333	0.079443	0.037756	0.020062	0.003628	0.042121	0.021916	0.017784										
12	**sAs_Afg**	0.044754	0.124665	0.097123	0.102102	0.052050	0.021304	0.008620	0.060075	0.035118	0.029735	0.001511									
13	**sEu**	0.064578	0.155621	0.119309	0.117913	0.066300	0.036737	0.015131	0.064593	0.039712	0.031804	0.003921	0.002064								
14	**eEu**	0.066210	0.158879	0.122818	0.122007	0.069013	0.037348	0.016296	0.067829	0.042182	0.034056	0.004511	0.002175	0.000038							
15	**wEu**	0.068642	0.163151	0.126834	0.126154	0.072004	0.038764	0.017691	0.070734	0.044494	0.036072	0.005254	0.002530	0.000130	0.000031						
16	**eAs**	0.112148	0.157404	0.093422	0.056769	0.057067	0.119908	0.055200	0.015755	0.021559	0.017606	0.060443	0.079037	0.071492	0.074740	0.077050					
17	**wAs_Cau**	0.114150	0.162684	0.097673	0.060689	0.059434	0.120082	0.054782	0.017359	0.022368	0.017846	0.058754	0.076677	0.068473	0.071609	0.073799	0.000099				
18	**nEu**	0.079650	0.181776	0.143916	0.143304	0.084726	0.045716	0.023990	0.082505	0.054067	0.044384	0.008885	0.004708	0.001084	0.000751	0.000480	0.085835	0.082097			
19	**nAf_Egt**	0.087395	0.174577	0.121158	0.102434	0.066203	0.066198	0.023311	0.043045	0.028058	0.019145	0.013337	0.017263	0.009810	0.010731	0.011240	0.032074	0.029501	0.013382		
20	**seAs**	0.098593	0.178065	0.117995	0.091480	0.065434	0.083794	0.031293	0.033343	0.024411	0.015989	0.023986	0.031810	0.022755	0.024282	0.025180	0.016697	0.014669	0.028655	0.002821	

**Fig 1:**
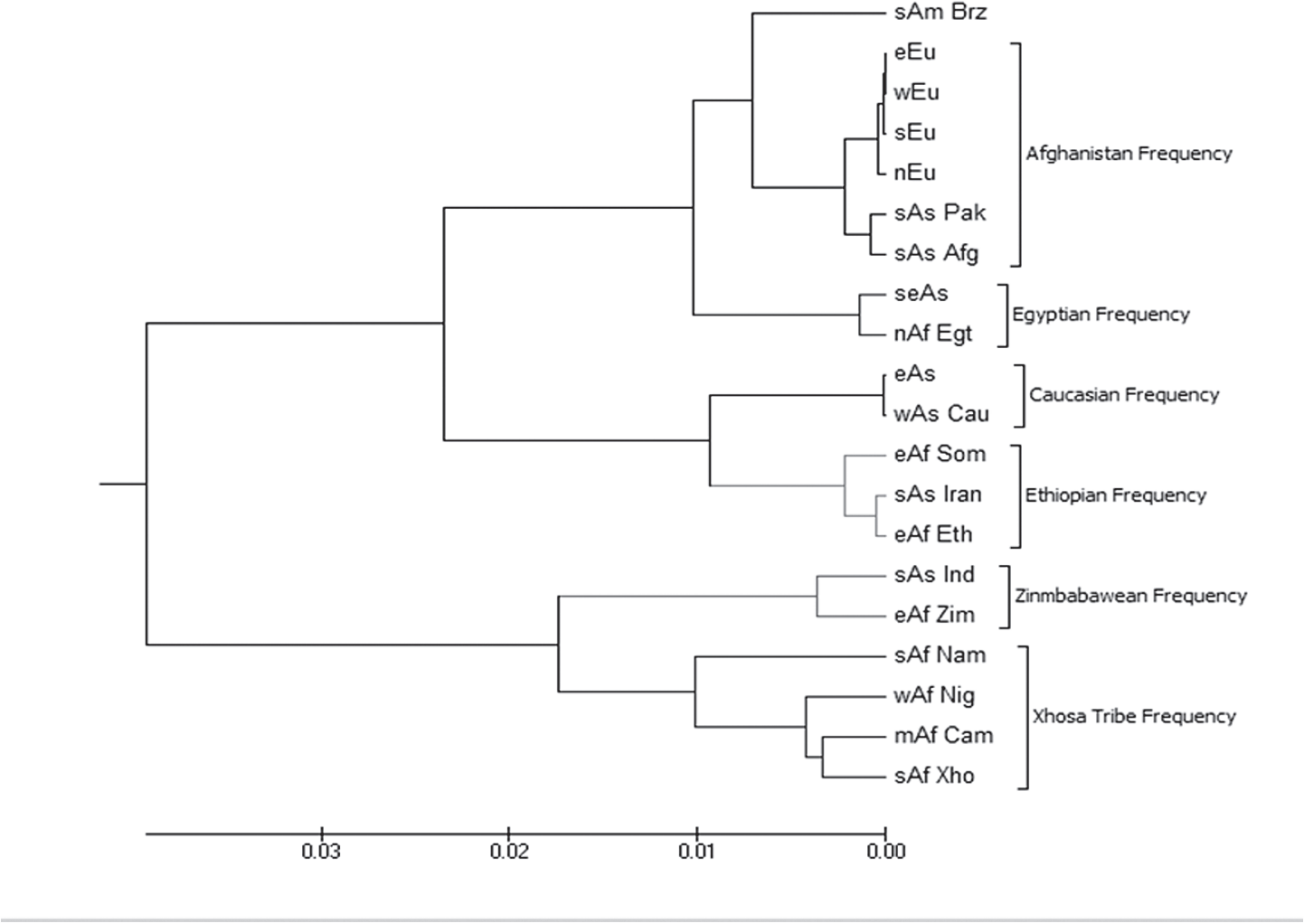
Phylogenetic tree of 20 different continental regions population affiliations for *GST M1-T1 null* allele frequency. The tree was produced by the UPGMA method from D_ST_ values in Table 2 and cluster with more than 50% of 1000 bootstrap replicates were included in the consensus tree obtained by Felsenstein (1989) phylogeny interference package. Major group of *GST M1-T1 null* allele frequencies were from population of Xhosa tribe, Zimbabwe, Ethiopia, Egypt, Afghanistan and Caucasian. Abbreviations used were same as those in Table 1.

The values represented in the table were computed between the population affiliations by Nei’s (1972) standard genetic distance (D_ST_) method and were used in phylogenetic tree of 20 different continental regions population affiliations for *GST M1-T1 null* allele frequency (Fig. 1). Abbreviations used were same as those in Table 1.

**Table 3:** *GST M1-T1 null* allele frequency based genetic distance between 45 geographically assorted human populations (GAHPs).

S. No.	GAHPs	eAf_Zim	sAf_Nam	Ind_Guj	sAf_Xho	Ind_Tn	Ind_Kar	Ind_Wb	mAf_Cam	Ind_Mah	wAf_Nig	Ind_Ker	Ind_Up	Ind_Ap	sAm_Brz
1	**eAf_Zim**														
2	**sAf_Nam**	0.019641													
3	**Ind_Guj**	0.008566	0.004982												
4	**sAf_Xho**	0.018002	0.007702	0.002144											
5	**Ind_Tn**	0.003500	0.033450	0.022055	0.037140										
6	**Ind_Kar**	0.003729	0.037771	0.023632	0.038408	0.000355									
7	**Ind_Wb**	0.012420	0.058122	0.041785	0.061245	0.003252	0.002526								
8	**mAf_Cam**	0.036741	0.028132	0.014999	0.006642	0.063507	0.062461	0.090338							
9	**Ind_Mah**	0.013628	0.064129	0.044263	0.063067	0.004934	0.003307	0.000574	0.088816						
10	**wAf_Nig**	0.011698	0.025018	0.007718	0.007576	0.026876	0.025085	0.042346	0.009135	0.040441					
11	**Ind_Ker**	0.018509	0.075817	0.052630	0.072183	0.008654	0.006243	0.002038	0.096709	0.000540	0.045207				
12	**Ind_Up**	0.011897	0.062884	0.039784	0.055683	0.006375	0.003826	0.003100	0.075196	0.001229	0.031146	0.001306			
13	**Ind_Ap**	0.011155	0.056511	0.029903	0.039082	0.013492	0.009762	0.014788	0.047057	0.011066	0.014791	0.011238	0.005068		
14	**sAm_Brz**	0.019686	0.071517	0.039623	0.047429	0.023229	0.018123	0.023228	0.050077	0.017729	0.017980	0.016579	0.009579	0.001318	
15	**eAf_Som**	0.047449	0.069856	0.039132	0.031371	0.071354	0.065680	0.087771	0.014241	0.080505	0.013198	0.082462	0.062745	0.031855	0.027019
16	**sAs_Iran**	0.033673	0.067958	0.035776	0.033278	0.050368	0.044624	0.060441	0.022080	0.053470	0.010096	0.054000	0.038840	0.015631	0.011558
17	**seAs_Vie**	0.027183	0.078906	0.043887	0.048612	0.034281	0.028238	0.035694	0.045118	0.028849	0.017602	0.027294	0.018065	0.004738	0.001318
18	**eAf_Eth**	0.041147	0.083845	0.047123	0.045069	0.056782	0.049923	0.064060	0.031565	0.055751	0.016495	0.054832	0.040296	0.016788	0.010910
19	**sAs_Pak**	0.037341	0.107807	0.067666	0.078333	0.036617	0.029669	0.029982	0.079443	0.022419	0.037756	0.018481	0.013929	0.007683	0.003628
20	**sAs_Afg**	0.044754	0.124665	0.082681	0.097123	0.039341	0.032172	0.028470	0.102102	0.020874	0.052050	0.015882	0.014104	0.012496	0.008620
21	**eAs_Mon**	0.041766	0.111479	0.069331	0.077606	0.043779	0.036213	0.038385	0.074231	0.029918	0.036336	0.025780	0.019540	0.009564	0.004097
22	**nEu_Fin**	0.056413	0.147166	0.103438	0.122646	0.045284	0.037978	0.029516	0.132931	0.022041	0.072547	0.015927	0.017497	0.021698	0.018273
23	**sEu_Ita**	0.061485	0.153594	0.106118	0.122436	0.053407	0.045069	0.038414	0.126364	0.029524	0.070064	0.022816	0.022503	0.022062	0.016456
24	**eEu_Rus**	0.059262	0.147482	0.099679	0.113354	0.054266	0.045694	0.041407	0.113525	0.032070	0.062336	0.025631	0.023486	0.019501	0.013120
25	**eAs_Jap**	0.110350	0.146520	0.099722	0.084594	0.142570	0.132315	0.158417	0.048140	0.144938	0.053266	0.143253	0.118214	0.072885	0.059379
26	**wEu_Ned**	0.059823	0.144141	0.095375	0.105798	0.058632	0.049635	0.048197	0.101024	0.038141	0.055834	0.031893	0.027464	0.018923	0.011362
27	**sEu_Spa**	0.060246	0.145849	0.097044	0.108186	0.058185	0.049224	0.047092	0.104342	0.037126	0.057711	0.030768	0.026857	0.019275	0.011877
28	**eEu_Pol**	0.065713	0.157598	0.107503	0.121115	0.060599	0.051493	0.046757	0.119726	0.036775	0.067624	0.029742	0.027621	0.023028	0.015739
29	**eEu_Slk**	0.067544	0.161727	0.111408	0.126171	0.061054	0.051968	0.046119	0.126173	0.036234	0.071687	0.028989	0.027678	0.024546	0.017452
30	**wEu_Ger**	0.070266	0.166596	0.115586	0.130954	0.063009	0.053801	0.047246	0.131318	0.037254	0.075319	0.029750	0.028878	0.026377	0.019164
31	**seAs_Phi**	0.070253	0.140613	0.090160	0.090823	0.080995	0.070962	0.078774	0.072213	0.066801	0.045336	0.061492	0.049833	0.027442	0.016743
32	**eEu_Bul**	0.072759	0.171473	0.120080	0.136502	0.064240	0.055011	0.047413	0.138030	0.037460	0.079764	0.029721	0.029583	0.028364	0.021260
33	**sEu_Sln**	0.066941	0.151257	0.099936	0.107876	0.068535	0.058783	0.059143	0.098202	0.048016	0.056728	0.041428	0.035289	0.022831	0.013657
34	**sEu_Gre**	0.084181	0.192766	0.140787	0.162602	0.069434	0.060400	0.047888	0.171015	0.038494	0.101709	0.029929	0.033419	0.038936	0.032905
35	**eAs_Kor**	0.131072	0.171461	0.120063	0.103009	0.165454	0.153899	0.180971	0.061388	0.165744	0.067925	0.162999	0.136593	0.087477	0.071632
36	**wAs_Cau**	0.114150	0.162684	0.110741	0.097673	0.142911	0.131528	0.153814	0.060689	0.139138	0.059434	0.135556	0.112713	0.069730	0.054782
37	**wEu_Fra**	0.079786	0.181720	0.127681	0.143431	0.071836	0.061962	0.054405	0.142357	0.043640	0.084247	0.035309	0.034781	0.032085	0.023807
38	**eAs_Chi**	0.107068	0.162350	0.109199	0.098764	0.132097	0.120617	0.139605	0.064352	0.125031	0.057523	0.120667	0.100165	0.060995	0.046306
39	**nEu_Dnk**	0.086463	0.194885	0.140372	0.159569	0.074495	0.064731	0.053988	0.162804	0.043567	0.097621	0.034687	0.036500	0.038115	0.030493
40	**nEu_Swd**	0.088823	0.197255	0.141108	0.158695	0.078575	0.068343	0.058687	0.158719	0.047608	0.096026	0.038515	0.039288	0.038475	0.029849
41	**nAf_Egt**	0.087395	0.174577	0.117293	0.121158	0.093461	0.082045	0.085005	0.102434	0.071600	0.066203	0.063981	0.055069	0.035784	0.023311
42	**seAs_Ids**	0.110068	0.176501	0.119385	0.111506	0.131239	0.118996	0.134139	0.077907	0.118852	0.064351	0.112960	0.094840	0.058800	0.043301
43	**eEu_Cze**	0.093352	0.195596	0.135981	0.146458	0.091159	0.079672	0.075757	0.134876	0.062724	0.084693	0.053635	0.049608	0.038780	0.027114
44	**nEu_Uk**	0.100862	0.209262	0.147632	0.159813	0.096473	0.084651	0.078719	0.149197	0.065400	0.094779	0.055589	0.052818	0.043805	0.031824
45	**seAs_S_M**	0.171196	0.272027	0.195663	0.189285	0.188418	0.171818	0.180613	0.146069	0.160261	0.120939	0.148990	0.133165	0.096023	0.074061

S. No.	GAHPs	eAf_Som	sAs_Iran	seAs_Vie	eAf_Eth	sAs_Pak	sAs_Afg	eAs_Mon	nEu_Fin	sEu_Ita	eEu_Rus	eAs_Jap	wEu_Ned	sEu_Spa	eEu_Pol	eEu_Slk	wEu_Ger
1	**eAf_Zim**																
2	**sAf_Nam**																
3	**Ind_Guj**																
4	**sAf_Xho**																
5	**Ind_Tn**																
6	**Ind_Kar**																
7	**Ind_Wb**																
8	**mAf_Cam**																
9	**Ind_Mah**																
10	**wAf_Nig**																
11	**Ind_Ker**																
12	**Ind_Up**																
13	**Ind_Ap**																
14	**sAm_Brz**																
15	**eAf_Som**																
16	**sAs_Iran**	0.003247															
17	**seAs_Vie**	0.018569	0.006249												
18	**eAf_Eth**	0.005520	0.000882	0.004922													
19	**sAs_Pak**	0.042121	0.021916	0.004849	0.017784												
20	**sAs_Afg**	0.060075	0.035118	0.011710	0.029735	0.001511											
21	**eAs_Mon**	0.034995	0.017298	0.003418	0.012829	0.000686	0.003668										
22	**nEu_Fin**	0.085813	0.054952	0.023795	0.048207	0.007250	0.002139	0.011285									
23	**sEu_Ita**	0.074994	0.047057	0.019441	0.039473	0.004902	0.001328	0.007153	0.001183								
24	**eEu_Rus**	0.063015	0.038137	0.014471	0.030855	0.002934	0.001176	0.003970	0.003238	0.000625							
25	**eAs_Jap**	0.013155	0.021518	0.042950	0.019082	0.068188	0.088912	0.055545	0.118879	0.099708	0.083923						
26	**wEu_Ned**	0.050728	0.029508	0.010569	0.022406	0.002541	0.002971	0.001993	0.007729	0.003189	0.000994	0.066381					
27	**sEu_Spa**	0.053620	0.031555	0.011506	0.024328	0.002600	0.002507	0.002372	0.006630	0.002461	0.000613	0.070139	0.000048				
28	**eEu_Pol**	0.065812	0.040687	0.016562	0.032638	0.004229	0.002209	0.004941	0.004040	0.000858	0.000165	0.083989	0.000992	0.000612			
29	**eEu_Slk**	0.071327	0.044829	0.018905	0.036525	0.005135	0.002279	0.006326	0.003107	0.000463	0.000305	0.090713	0.001792	0.001261	0.000119		
30	**wEu_Ger**	0.075125	0.047827	0.020825	0.039197	0.006090	0.002724	0.007459	0.002961	0.000465	0.000576	0.094485	0.002374	0.001762	0.000297	0.000046	
31	**seAs_Phi**	0.024328	0.014094	0.009674	0.007943	0.012859	0.020235	0.007606	0.033587	0.023306	0.016243	0.026061	0.009176	0.010551	0.015906	0.018775	0.020380
32	**eEu_Bul**	0.080715	0.052137	0.023456	0.043215	0.007353	0.003211	0.009134	0.002524	0.000471	0.001061	0.100979	0.003445	0.002695	0.000739	0.000272	0.000099
33	**sEu_Sln**	0.045379	0.026651	0.010844	0.019074	0.004789	0.006643	0.002802	0.013235	0.006789	0.003365	0.055063	0.000756	0.001124	0.002972	0.004248	0.004987
34	**sEu_Gre**	0.109969	0.075001	0.038093	0.065044	0.015588	0.007723	0.019615	0.002584	0.003053	0.006064	0.136499	0.011461	0.010027	0.005709	0.004178	0.003432
35	**eAs_Kor**	0.020729	0.030293	0.053132	0.026573	0.078627	0.099976	0.064572	0.130934	0.109591	0.092866	0.000844	0.074020	0.077986	0.092201	0.099211	0.102957
36	**wAs_Cau**	0.017359	0.022368	0.038777	0.017846	0.058754	0.076677	0.046671	0.103297	0.084432	0.070009	0.001457	0.053918	0.057268	0.069306	0.075314	0.078511
37	**wEu_Fra**	0.081468	0.053324	0.025048	0.043630	0.008767	0.004756	0.009914	0.004324	0.001326	0.001554	0.097852	0.003395	0.002726	0.000858	0.000491	0.000281
38	**eAs_Chi**	0.018032	0.019863	0.031772	0.014526	0.047768	0.063315	0.036879	0.086980	0.069532	0.056610	0.003945	0.042294	0.045231	0.055758	0.061090	0.063888
39	**nEu_Dnk**	0.099377	0.067166	0.033629	0.056781	0.013226	0.006821	0.015800	0.003522	0.002129	0.003908	0.119580	0.007674	0.006567	0.003182	0.002132	0.001552
40	**nEu_Swd**	0.093746	0.063203	0.031711	0.052536	0.012575	0.007061	0.014249	0.004890	0.002333	0.003353	0.110055	0.006111	0.005205	0.002389	0.001634	0.001153
41	**nAf_Egt**	0.043045	0.028058	0.016861	0.019145	0.013337	0.017263	0.008801	0.026617	0.016665	0.011225	0.041403	0.005917	0.006792	0.009951	0.011966	0.012884
42	**seAs_Ids**	0.024721	0.022959	0.029672	0.015914	0.040623	0.053406	0.030595	0.073751	0.057083	0.045637	0.009640	0.032974	0.035482	0.044243	0.048847	0.051121
43	**eEu_Cze**	0.068673	0.045834	0.024135	0.035150	0.011933	0.010842	0.010092	0.014146	0.007226	0.004934	0.072461	0.003447	0.003437	0.003425	0.004031	0.004140
44	**nEu_Uk**	0.079197	0.054143	0.029441	0.042558	0.014548	0.012026	0.013217	0.013662	0.007192	0.005662	0.082889	0.005059	0.004822	0.003909	0.004143	0.003995
45	**seAs_S_M**	0.065903	0.059196	0.058083	0.045957	0.059246	0.067676	0.048143	0.083276	0.063958	0.053621	0.034562	0.041689	0.043754	0.049723	0.053443	0.054630

S. No.	GAHPs	seAs_Phi	eEu_Bul	sEu_Sln	sEu_Gre	eAs_Kor	wAs_Cau	wEu_Fra	eAs_Chi	nEu_Dnk	nEu_Swd	nAf_Egt	seAs_Ids	eEu_Cze	nEu_Uk	seAs_S_M
1	**eAf_Zim**															
2	**sAf_Nam**															
3	**Ind_Guj**															
4	**sAf_Xho**															
5	**Ind_Tn**															
6	**Ind_Kar**															
7	**Ind_Wb**															
8	**mAf_Cam**															
9	**Ind_Mah**															
10	**wAf_Nig**															
11	**Ind_Ker**															
12	**Ind_Up**															
13	**Ind_Ap**															
14	**sAm_Brz**															
15	**eAf_Som**															
16	**sAs_Iran**															
17	**seAs_Vie**															
18	**eAf_Eth**															
19	**sAs_Pak**															
20	**sAs_Afg**															
21	**eAs_Mon**															
22	**nEu_Fin**															
23	**sEu_Ita**															
24	**eEu_Rus**															
25	**eAs_Jap**															
26	**wEu_Ned**															
27	**sEu_Spa**															
28	**eEu_Pol**															
29	**eEu_Slk**															
30	**wEu_Ger**															
31	**seAs_Phi**															
32	**eEu_Bul**	0.023307														
33	**sEu_Sln**	0.005174	0.006459													
34	**sEu_Gre**	0.040845	0.002371	0.016708												
35	**eAs_Kor**	0.030312	0.109659	0.061189	0.146691											
36	**wAs_Cau**	0.018205	0.084286	0.043046	0.116556	0.001522										
37	**wEu_Fra**	0.021768	0.000244	0.005702	0.003136	0.105513	0.080637									
38	**eAs_Chi**	0.011841	0.069030	0.032590	0.098141	0.004400	0.000757	0.065569								
39	**nEu_Dnk**	0.032106	0.000897	0.011395	0.000743	0.128153	0.100281	0.000977	0.083214							
40	**nEu_Swd**	0.027511	0.000747	0.008887	0.001958	0.117637	0.091120	0.000396	0.074860	0.000289						
41	**nAf_Egt**	0.002571	0.014962	0.002462	0.028758	0.044664	0.029501	0.012785	0.020733	0.020707	0.016505					
42	**seAs_Ids**	0.007667	0.055554	0.024043	0.081357	0.009757	0.003788	0.051849	0.001246	0.067380	0.059583	0.012972				
43	**eEu_Cze**	0.011933	0.004923	0.002625	0.012358	0.076943	0.056281	0.003219	0.043721	0.007079	0.004562	0.004116	0.031688			
44	**nEu_Uk**	0.016414	0.004424	0.004695	0.010232	0.087293	0.065142	0.002633	0.051494	0.005446	0.003224	0.006718	0.038033	0.000355		
45	**seAs_S_M**	0.019871	0.058065	0.031312	0.080243	0.030458	0.021755	0.051916	0.016477	0.065050	0.056451	0.016134	0.009626	0.028910	0.032338	

**Fig 2:**
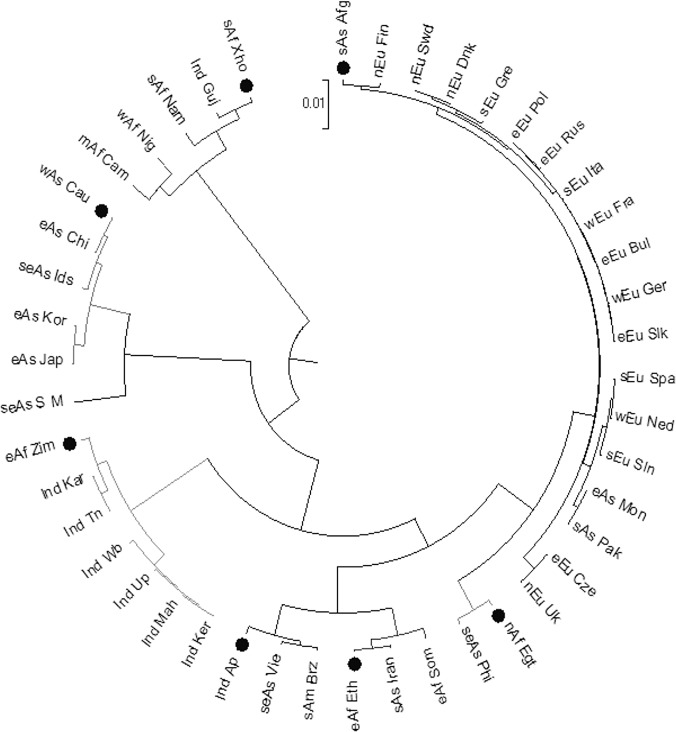
Phylogenetic tree of 45 geographically assorted human populations for *GST M1-T1 null* allele frequency. This tree was based on D_ST_ values in Table 3. Other aspects were same as those in Fig. 1. Major group of *GST M1-T1 null* allele frequencies were from population of Xhosa tribe, Zimbabwe, Ethiopia, Egypt, Afghanistan, Caucasian and Andhra Pradesh. Abbreviations used were same as those in Table 1.

The values represented in the table were computed between the population affiliations by Nei’s (1972) standard genetic distance (DST) method and were used in phylogenetic tree of 45 geographically assorted human populations for *GST M1-T1 null* allele frequency (Fig. 2). Abbreviations used were same as those in Table 1.

### Statistical analysis

Distributions of *GST M1* and *GST T1 null* alleles in Gujarati population were calculated by frequency counting method in SPSS-21 (4-27AEA) for windows. The standard genetic distance (D_ST_) between different PAs for *GST M1-T1 null* allele frequencies were calculated by Nei’s (1972) method in Phylip 3.69 version [52, 53]. Least D_ST_ values between the PAs were used to compute clades with more than 50% of 1000 bootstrap replicates by Felsenstein (1989) method and then the phylogenetic trees were constructed in Mega-6 software by UPGMA method [54–56]. Finally, the clusters of PAs split found among the geographically assorted human populations in phylogenetic tree were used in the scattered plot to analyze their geographical distribution. The longitude (X-axis) and latitude (Ys-axis) of different continental regions were used to construct the scattered plot in SPSS-21 as summarized in Table 4. The online web source world atlas was used to compute the latitude and longitude of the respective geographic locations (http://www.worldatlas.com/aatlas/latitude_and_longitude_finder.htm [57]).

**Table 4:** Geographical location of population affiliations from 20 different continental regions used in scattered plot analyses.

Location ^a^	Latitude	Longitude
sAf_Xho	- 30.33	22.56
eAf_Zim	- 19.0	29.9
nAf_Egp	26.49	30.48
eAf_Eth	9.8	40.29
wAf_Nig	9.4	8.4
mAf_Cam	7.22	12.21
eAf_Som	5.9	46.11
sAf_Nam	- 22.57	18.29
sAs_Iran	32.25	53.41
sAs_Pak	30.22	69.20
sAs_Af	33.56	67.42
sAs_Ind	20.35	78.57
eAs	22.16	114.14
seAs	11.35	121.37
wAs_Cau	43	43.45
nEu	62.16	12.20
wEu	46.12	1.15
sEu	41.16	- 1.12
eEu	59.80	36.29
sAm _Brz	-14.14	- 51.55

^a^ Latitudes in the northern hemisphere were listed with positive values, as were longitudes in the eastern hemisphere; Latitudes in the southern hemisphere were listed with negative values, as in longitudes of western hemisphere. Abbreviations used were same as those in Table 1.

## Results

### Phylogenetic tree for *GST M1-T1 null* allele frequency in GAHPs

The frequency of *GST M1* and *T1 null* genotypes in Gujarat populations of India was observed as 0.200 and 0.355 respectively in this present study. The pair wise genetic distance matrix computed for *GST M1*-*T1 null* allele frequency between the respective PAs using Nei’s (1972) standard genetic distance method (D_ST_) in gendist program of Phylip-3.69 software for the 20 different continental regions PAs and the 45 GAHPs described elsewhere were summarized in Tables 2 and 3 respectively. The phylogenetic analyses of 20 different continental regions (Fig. 1) and 45 GAHPs (Fig. 2) for *GST M1*-*T1 null* allele frequency were performed using the pair wise genetic distance matrix by UPGMA method in MEGA-6 software [52, 56]. Consense program that clustered more than 50% of 1000 bootstrap replicates was used to assess the reliability of the constructed phylogenetic trees [53, 54]. The Nei’s D_ST_ value varies from 0.0001 to 0.007 (Table 2) and 0.0006 to 0.008 (Table 3) for *GST M1-T1 null* allele frequency among the 20 different continental regions PAs and the 45 GAHPs respectively. The phylogenetic trees in Figs. 1 and 2 showed consistent clusters or patterns for *GST M1-T1 null* allele frequency from populations such as South African Xhosa tribe, South African Zimbabwe, East African Ethiopia, North African Egypt, Caucasian and South Asian Afghanistan. The least D_ST_ value of PAs from South Asian Iran (0.000882) and East African Somalia (0.00552) with *East African Ethiopian* allele frequency grouped them as one of the clusters in the phylogenetic trees for *GST M1-T1 null* allele frequency. Few of the African PAs such as West African Nigeria, Middle African Cameroon and South African Namibia were clustered with *South African Xhosa* tribe allele frequency with least D_ST_ value that ranged from 0.006642 to 0.007702. In addition, the South Asian Indian Gujarat PAs (0.00214) was observed with highest affinity to South African Xhosa cluster than any other clusters (Fig. 2). The average *GST M1-T1 null* allele frequency in South Asian Indian PA was observed as a cluster to *East African Zimbabwe* allele frequency (Fig. 1) with least D_ST_ value (0.007203) out of 20 continental regions. Further, the least D_ST_ value to East African Zimbabwe allele frequency was observed with South Asian Indian PAs (Table 3) from Tamilnadu (0.0035) and Karnataka (0.003729). Furthermore, PA from Karnataka was observed with least D_ST_ value to Maharashtra allele frequency (0.003307), which was clustered with allele frequency from West Bengal (0.000574), Uttar Pradesh (0.003826) and Kerala PAs of South Asian India (0.006243) as shown in Fig. 2. Nevertheless, the *South Asian Indian Andhra Pradesh* allele frequency was observed with least D_ST_ value only to Uttar Pradesh PA (0.005068) than any other Indian PAs for *GST M1-T1* null allele frequency and stood as a separate cluster in the phylogenetic tree with highest genetic affinity to South America (Brazil, 0.001318) and South East Asia allele frequency (Vietnam, 0.004738) from geographically distant continental regions.

PAs from China (0.000757), Japan (0.001457) and Korea (0.001522) of East Asia and Pakistan (0.001511) of South Asia were observed as another cluster with least D_ST_ value to *Caucasians* [Americans and Canadians (10)] for *GST M1-T1 null* allele frequency out of 45 combinations (Fig. 2). However, Mongolia of East Asia was observed with least D_ST_ value to Pakistan allele frequency (0.000686) than the Caucasians (0.046671). The least D_ST_ value between European continental regions and *South Asian Afghanistan* allele frequency that ranged from 0.002064 to 0.004708 was clustered together for *GST M1-T1 null* allele frequency in the phylogenetic tree of 20 different continental regions (Fig. 1). Nevertheless, the phylogenetic tree of 45 GAHPs (Fig. 2) clustered only 13 European PAs (Sweden, Finland, Denmark, Netherlands, Germany, France, Italy, Spain, Greece, Bulgaria, Poland, Slovakia and Russia) out of 16 investigated in this study with South Asian Afghanistan allele frequency (least D_ST_ value that ranged from 0.001176 to 0.00723) while, the other 3 PAs [Slovenia (0.002462), Czech Republic (0.004116) and UK (0.006718)] were clustered with *North African Egypt* allele frequency. Singapore-Malay and Indonesia PAs from South East Asia were observed with least D_ST_ value to East Asia (China, 0.001246) and Caucasian (0.003788) respectively for *GST M1-T1 null* allele frequency. Nevertheless, the other counter parts from same continental region were observed as the most diverse PAs with PA admixture from North Africa (Egypt, 0.002571) and East Africa (Ethiopia, 0.007943) for Philippines; South Asia India (Andhra Pradesh, 0.004738) and East Africa (Ethiopia, 0.004922) for Vietnam among the 45 GAHPs investigated in this study for *GST M1-T1 null* allele frequency as shown in Table 3 and Fig. 2 respectively.

### *GST M1-T1 null* allele frequency patterns among the GAHPs

**Fig 3:**
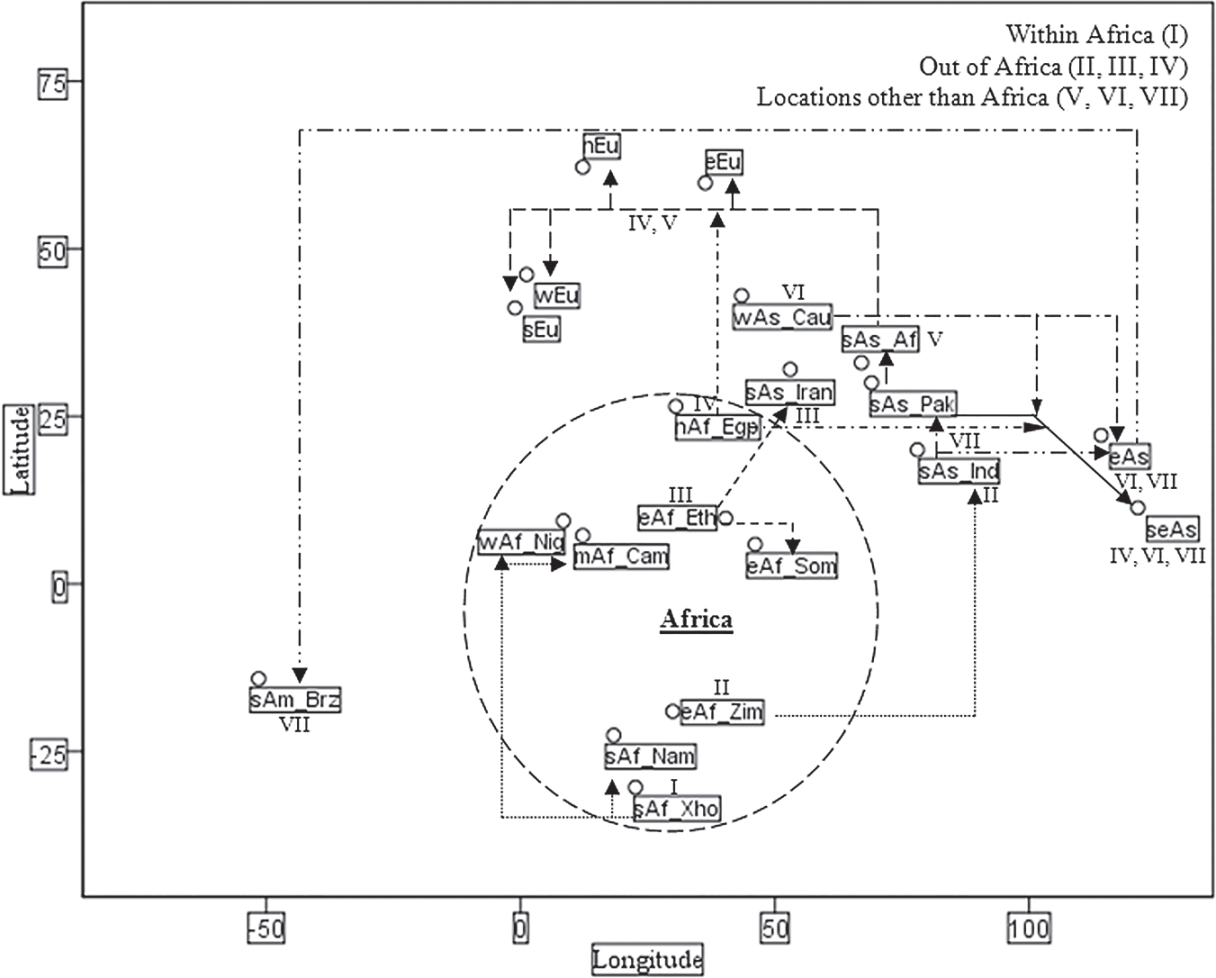
Geographic Location of *GST M1-T1 null* allele frequency patterns in Scattered Plot. This scenario is largely a speculation based on Nei’s D_ST_ based phylogenetic trees in Figs. 1 and 2, which suggest seven patterns for *GST M1*-*T1* null allele frequency among the 45 geographically assorted human populations. The scattered plot shows the genetic affinity and geographical distribution of the seven probable patterns with three major split among the continental regions such as an “African” split with South African Xhosa pattern (I), an “out of Africa” split with East African Zimbabwe (II), East African Ethiopia (III), North African Egypt (IV) patterns and an “other than Africa” split with South Asian Afghanistan (V), West Asian Caucasian (VI), South Asian Indian Andhra Pradesh patterns (VII). Abbreviations used were same as those in Table 1.

The effect of isolation by geographical distance in population differentiation [51] was validated in a scattered plot with respect to the phylogenetic clusters of 45 GAHPs for *GST M1-T1 null* allele frequency that corresponds to the latitudes and longitudes of 20 different continental regions representing PAs from Africa, Asia, Europe and America (Table 4). The scattered plot illustrated in Fig. 3 suggest three major geographical split for the seven *GST M1-T1 null* allele frequency clusters or patterns observed in the phylogenetic tree of 45 GAHPs (Fig. 2). South African Xhosa allele frequency pattern (I) observed mostly in continental regions of Africa suggest an “Africa” split in the scattered plot with least population differentiation to Nigeria (West Africa), Cameroon (Middle Africa) and Namibia (South Africa). However, the *GST M1-T1 null* allele frequency patterns of PAs from other African continental region such as East African Zimbabwe (II), East African Ethiopia (III) and North African Egypt (IV) were observed with least population differentiation to PAs from non African continental regions such as South Asia (India and Iran), South East Asia (Philippines and Vietnam), Southern Europe (Slovenia), Eastern Europe (Czech Republic) and Northern Europe (UK) irrespective of the geographical isolation suggest an “out of Africa” split. Finally, the remaining three non-African *GST M1-T1 null* allele frequency patterns observed from Caucasian (V), South Asian Afghanistan (VI) and South Asian Indian Andhra Pradesh (VII) were geographically distributed in different continental regions such as Asia, Europe and America with the exception of Africa suggest an “other than Africa” split among the 45 GAHPs in the scattered plot.

## Discussion

Understanding the genetic variation and diversity among the geographically assorted human populations (GAHPs) is an interesting topic in population genetics with a wide range of neutral genetic markers and adaptive markers being employed to uncover the patterns of genetic diversity [58]. To address the extent of diversity in allele frequency distribution among populations from different ethnicity, region, country or continent is difficult, nevertheless understanding this phenomenon is inevitable [8, 11, 16, 17] and recent advances in the molecular techniques excel the perspective of inter-individual genetic variations in GAHPs. Allele frequencies of large number of neutral markers or of even few candidate markers that duplicate or decay to favor new environments and lead to rapid adaptations are often used for investigating the patterns [58]. The paradigm of allele frequency among the populations holds the key to unlock the existing problem of inter-individual genetic variation in xenobiotic metabolizing enzymes (XMEs) and in particular the decay or null allele frequency of Glutathione-S-transferase’s classes such as Mu 1 (*GST M1*) and Theta 1 (*GST T1*), which are considered as the major risk factor for various diseases including several types of cancers [8–10]. Therefore, the present investigation analyzed the pattern for *GST M1-T1 null* allele frequency among GAHPs using a phylogenetic approach. A set of 20 different continental region PAs (Table 2) and 45 GAHPs (Table 3) were recruited for *GST M1-T1 null* allele frequency data from 38 previously reported works and genomic data of Indian Gujarat PA in this study (Table 1) and the respective phylogenetic trees (Figs. 1 and 2) have been constructed by UPGMA method based on Nei’s (1972) standard genetic distance (D_ST_) with clusters more than 50% of 1000 bootstrap replicates obtained by Felsenstein (1989) program [52, 54, 56]. In addition to the ancestral origin or genetic affinity based clusters for *GST M1-T1 null* allele frequency demonstrated in the phylogenetic trees, a positive correlation between the genetic distance and geographical distance were analyzed for the effect of isolation in population differentiation by distance in a scattered plot (Fig. 3). Indeed, the observations from phylogenetic trees and scattered plot of different PAs constructively reveals the findings of seven probable patterns for *GST M1-T1 null* allele frequency among the GAHPs in concordance to the reports of archeological signatures, ancient gene flows and sex-specific components [59–62].

The genetic affinity and geographical distribution of 20 different continental regions that included 45 GAHPs investigated in this study (Figs. 1–3) revealed the findings of an allele frequency pattern for *GST M1-T1 null* genotypes among Namibia (South Africa), Nigeria (West Africa), Cameroon (Middle Africa), Gujarat (South Asian Indian) and Xhosa tribe (South Africa) for the first time. We report here, the findings of Xhosa allele frequency (I) with major genetic affinity towards populations from Africa (Namibia, Nigeria, Cameroon) as an “Africa” split pattern for *GST M1-T1 null* allele frequency in agreement to the reports of linkage disequilibrium computed for loss of variants in *GST* classes by Polimanti et al. (2013). The observations in phylogenetic trees (Figs. 1 and 2) and scattered plot analysis (Fig. 3) demonstrated the findings of another three patterns such as East Africa Zimbabwean allele frequency - II in population from India (South Asia), East Africa Ethiopian allele frequency - III in populations from Iran (South Asia) and Somalia (East Africa) and North Africa Egyptian allele frequency - IV in populations from Slovenia (Southern Europe), Czech Republic (Eastern Europe) and UK (Northern Europe) for *GST M1-T1 null* genotypes. The findings of Ethiopian - III and Egyptian - IV allele frequency pattern for *GST M1-T1 null* genotypes in this study are in concordance to the earlier reports of genome wide diversity study in the Levant by Haber et al., (2013), who found two major groups with one close to Africans and Middle Easterners and the other closer to modern day Europeans [61]. Further, the findings of these Zimbabwean, Ethiopian and Egyptian patterns from African populations with high genetic affinity towards non-African populations for *GST M1-T1 null* allele frequency have been reported as an “Out of Africa” split in this study in corroborate to the findings of Templeton (2002), who reported the out-of-Africa theory of migration and the ancestral root of allele frequency admixture [63].

The allele frequency of population from South Asian Afghanistan with high genetic affinity to majority of European PAs investigated in this study (Tables 2 and 3 and Figs. 2 and 3) has been reported as pattern - V for *GST M1-T1 null* genotypes in accordance to the reports of various authors [60, 62, 64]. Further, population in Pakistan (South Asia) has been reported with Afghanistan (South Asia) pattern for *GST M1-T1 null* allele frequency (Table 3) in this study, though it was found with genetic affinity to PAs from Mongolia (East Asia), Europe (South, East and West) and Andhra Pradesh (South India) in corroborate to the earlier reports of Templeton (2002), who stated the findings of considerable overlap among East Asians, Europeans and South Indian populations [64]. Moreover, the pattern of allele frequency from Caucasians (Americans and Canadians) found among East Asians (Fig. 1) in this study has been identified as pattern - VI for *GST M1-T1 null* allele frequency and reported for the first time. Finally, the allele frequency from South Indian Andhra Pradesh PA was found with least genetic distance (Table 3) to populations from Pakistan (South Asia), Vietnam (South East Asia) and Brazil (South America) irrespective of the phenomenon of population differentiation by geographical isolation [52] and has been reported as pattern - VII for *GST M1-T1 null* genotypes (Figs. 2 and 3) among the 45 GAHPs in this study. These observations of South East Asian and South American PAs with the *null* allele frequency pattern from South Indian Andhra Pradesh PA are in agreement to the reports of agro-pastoral system in South India that acted as agricultural center and source of dispersion to lineages from different preexisting populations [60]. Furthermore, the reported East Africa patterns from Zimbabwe (II), Ethiopia (III) among India, Iran (South Asia) and South Asian pattern from South Indian Andhra Pradesh (VII) among Vietnam (South East Asia) populations in this study for *GST M1-T1 null* allele frequency are in concordance to the reports of migration pattern of Homo sapiens from East Africa with the demographic expansions by Field and Lahr (2006), who investigated the geographic information systems during oxygen isotope stage 4 [62]. *GST M1-T1 null* allele frequency from South East Asian PAs has been reported as the complex admixture of Zimbabwe (II), Ethiopia (III) and Andhra Pradesh (VII) patterns in this study. Finally, the scattered plot analysis (Fig. 3), clearly demonstrates the findings of allele frequency patterns from South Asian Afghanistan - V, Caucasian - VI and South Indian Andhra Pradesh - VII as an “Other than Africa” split among 45 GAHPs for *GST M1-T1 null* genotypes with respect to their geographical distribution. This observation of other than Africa split in this study has been reported here in agreement to the concepts of later migration of the populations in regions other than Africa [60, 64]. In conclusion, the data of seven patterns for *GST M1-T1 null* allele frequency from Xhosa tribe (I), Zimbabwe (II), Ethiopia (III), Egypt (IV), Afghanistan (V), Caucasian (VI) and South Indian Andhra Pradesh (VII) reported in this study compare constructively with the earlier studies that suggested the PAs of relatively recent origin show comparatively small genetic differences and high genetic affinity among them [11, 46, 52, 58]. Findings of these seven patterns (I-VII) for *GST M1-T1 null* allele frequency reported here, would shed some light to address the missing link in most of the genomic epidemiological studies that lacks conclusive risk association [9, 16, 17]. The “Africa” (I), “Out of Africa” (II, III and IV) and “Other than Africa” (V, VI and VII) split among the 45 GAHPs reported in this study have to be explored further to rationalize the *GST M1-T1 null* allele’s frequency patterns in world populations.
